# Supplementary material for: Inverse centrifugal effect induced by collective motion of vortices in rotating thermal convection
Source: Nat Commun. 2021 Sep 22;12:5585. doi: 10.1038/s41467-021-25838-3 (PMC8458392; doi:10.1038/s41467-021-25838-3)
Supplement: Supplementary file 1 — Supplementary Information [file 41467_2021_25838_MOESM1_ESM.pdf]

Supplementary information for
Inverse centrifugal effect induced by collective motion of vortices in rotating thermal convection

Shan-Shan Ding[1], Kai Leong Chong[2], Jun-Qiang Shi[1], Guang-Yu Ding[2,3], Hao-Yuan Lu[1], Ke-Qing Xia*[3,2], and Jin-Qiang Zhong†[1]

[1]School of Physics Science and Engineering, Tongji University, Shanghai 200092, China.

[2]Department of Physics, The Chinese University of Hong Kong, Shatin, Hong Kong, China.

[3]Center for Complex Flows and Soft Matter Research and Department of Mechanics and Aerospace Engineering, Southern University of Science and Technology, Shenzhen 518055, China.

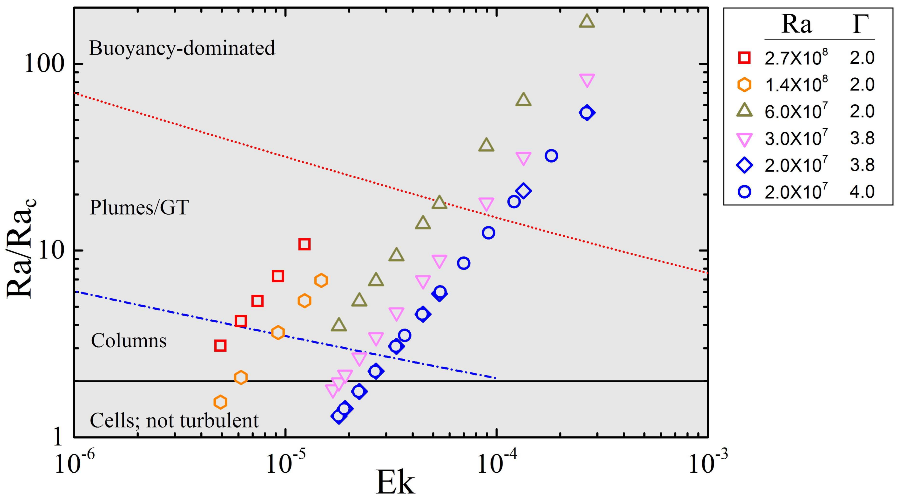

Supplementary Figure 1: Phase diagram of rotating convection and the parameters used in the present study. The parameter space ($\mathrm{Ek}, \mathrm{Ra}/\mathrm{Ra_c}$) is divided into four convection states, i.e., Buoyancy-dominated, Plumes/Geostrophic Turbulence (GT), Columns and Cells. Dotted red line ($\mathrm{Ra}=3.4\mathrm{Ek}^{-1.7}$): scaling for the transition between buoyancy-dominated convection and geostrophic turbulence, as suggested in [3, 4]. Dash-dotted blue line ($\mathrm{Ra}=1.18\mathrm{Ek}^{-1.6}$): transition between Plume/GT and Columns [4]. Solid black line ($\mathrm{Ra}/\mathrm{Ra_c}=2$): transition between Columns and Cells [1, 2, 3].

*xiakq@sustech.edu.cn
†jinqiang@tongji.edu.cn

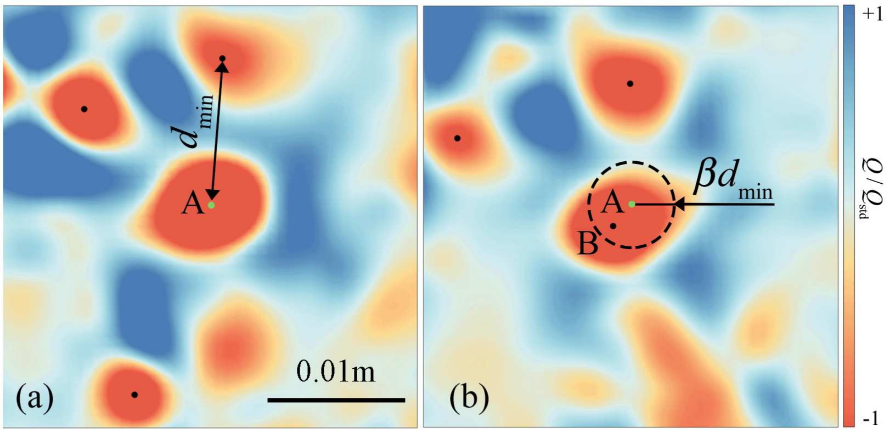

Supplementary Figure 2: Illustration of the scheme used in tracking a vortex. (a) Vortex A is identified with its centroid denoted by a green dot. (b) In the next frame, vortex B found in the immediate vicinity of A (within the dashed circle) is considered to be the same vortex. The background color represents the Q-value distribution.

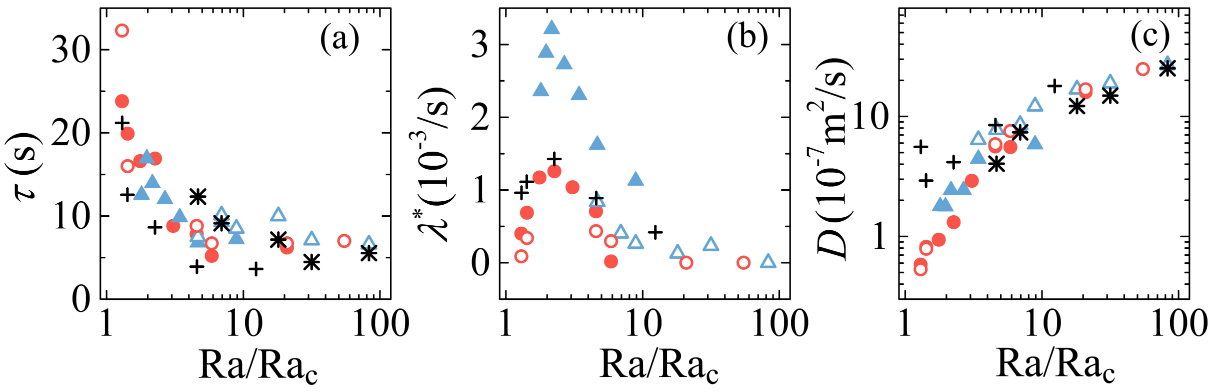

Supplementary Figure 3: Parameters of the vortex motion as functions of Ra/Ra_c. (a) momentum relaxation timescale τ, (b) the fastest growth (slowest decay) rate λ^* for anticyclones (cyclones) and (c) diffusivity D. Results for $Ra=2.0\times10^7$ (circle) and $Ra=3.0\times10^7$ (triangle). Open symbols: cyclones; filled symbols: anticyclones; pluses: DNS data for $Ra=2.0\times10^7$. Data of τ and D for $Ra=3.0\times10^7$ obtained through normal-diffusion fitting (Supplementary Eq. 14) of the experimental data in the randomly-diffusive regime are marked with stars. As the model (Supplementary Eqs. 3 and 4) cannot explain the inverse centrifugal motion of the cyclones, no fitting is made for their motion in the anomalous regime.

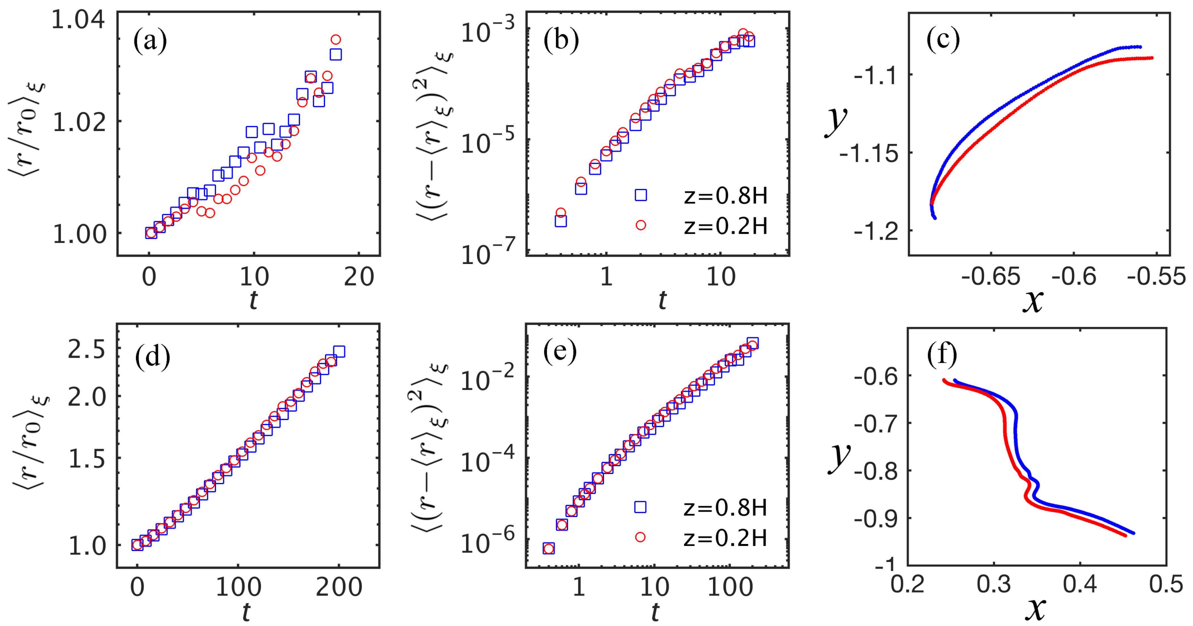

Supplementary Figure 4: DNS data for the first and second moments of radial vortex displacements. Data are for warm (a, b) and cold (d, e) vortices. Red circles: data for z=0.2H. Blue squares: data for z=0.8H. Individual vortex trajectories are shown in the Cartesian coordinate in (c) for a warm vortex, and in (f) for a cold vortex. Ra=2.0×10^7, Ra/Ra$_c$=1.76 and Fr=0.174.

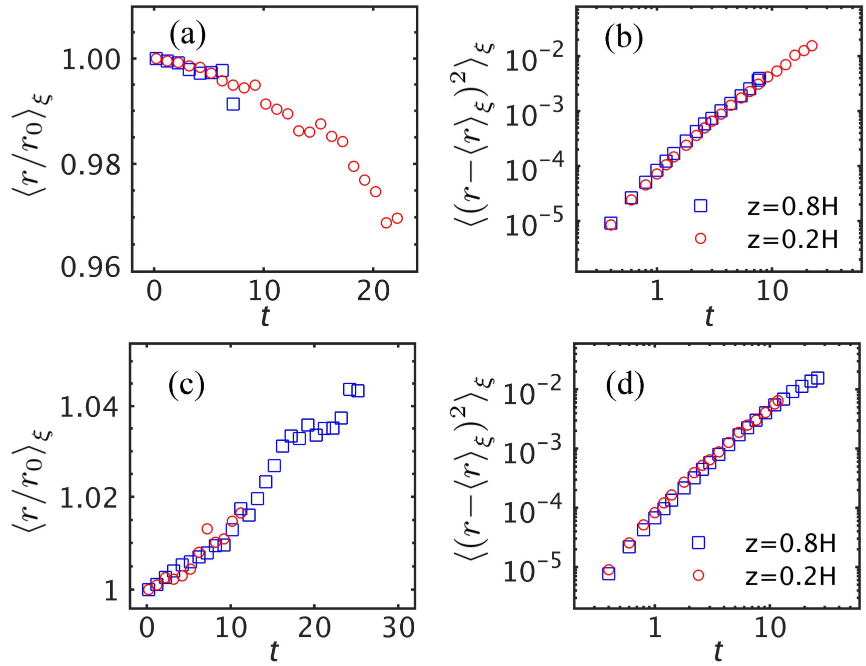

Supplementary Figure 5: DNS data for the first and second moment of radial displacements of the vortices. Results for warm (a,b) and cold (c, d) vortices are shown. Red circles: data for z=0.2H. Blue squares: data for z=0.8H. Results for Ra=2.0×10^7, Ra/Ra$_c$=5.99 and Fr=0.029.

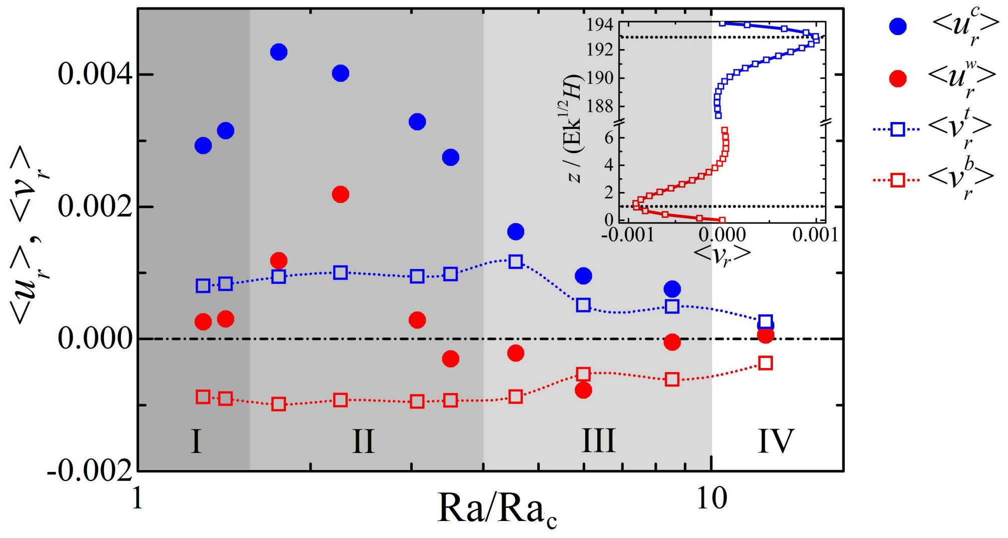

Supplementary Figure 6: The mean radial velocities $\langle u_r \rangle$ of the vortices (solid symbols) and of the fluid circulations $\langle v_r \rangle$ near the boundaries (open symbols) as functions of Ra/Ra$_\mathrm{c}$. Superscripts c and w are for cold and warm vortices, and superscripts t and p are circulations near the top and bottom boundaries, respectively. Data are taken at a radial position r=$d/4$. To represent the strength of the meridional circulations, the fluid velocities are determined at distances z=Ek$^{1/2}H$ from the top and bottom plates, where the magnitude of $\langle v_r \rangle$ is maximum (see inset). The background color indicates the four different regimes of vortex motions. Inset: vertical profiles of $\langle v_r \rangle$ near the top and bottom boundaries. Results for Ra/Ra$_\mathrm{c}$=2.26. Note that in regime IV, the vortices perform Brownian motion and therefore they do not exhibit a noticeable radial velocity.

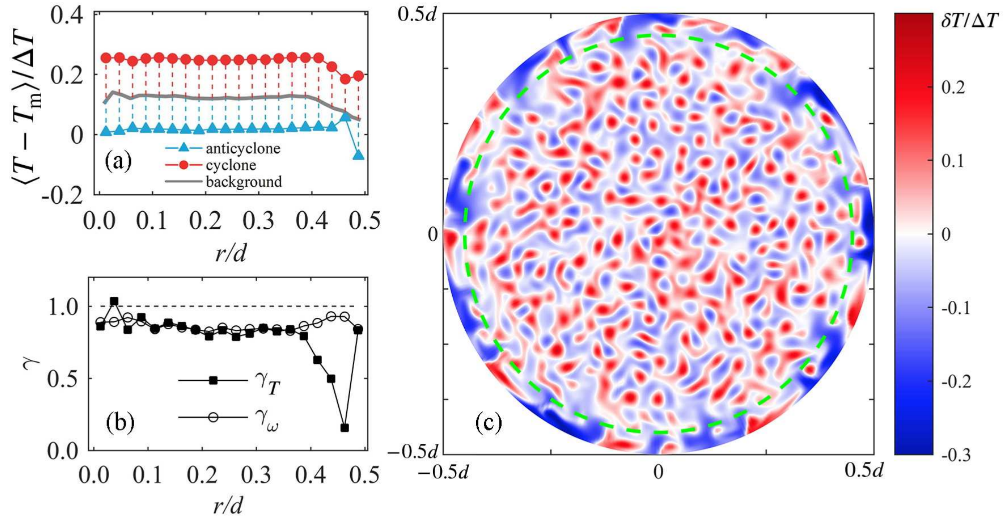

Supplementary Figure 7: Numerical results of the flow fields with the centrifugal buoyancy turned-off. (a) Radial profiles of the mean temperatures $\langle T - T_\mathrm{m} \rangle/\Delta T$ for cyclones (red circles), anticyclones (blue triangles) and the background fluid (solid line). The length of the dashed lines indicates the temperature difference δT between the cyclones (anticyclones) and the background fluid. (b) Radial profiles of of γ_ω and γ_T. (c) Distribution of the temperature anomaly $\delta T/\Delta T$. The green dashed circle denotes a radial position r/d=0.45 where temperature fluctuations due to the boundary flows are dominant. DNS data obtained at z=0.2H for Ra=2.0×10^7 and Ra/Ra$_\mathrm{c}$=2.26.

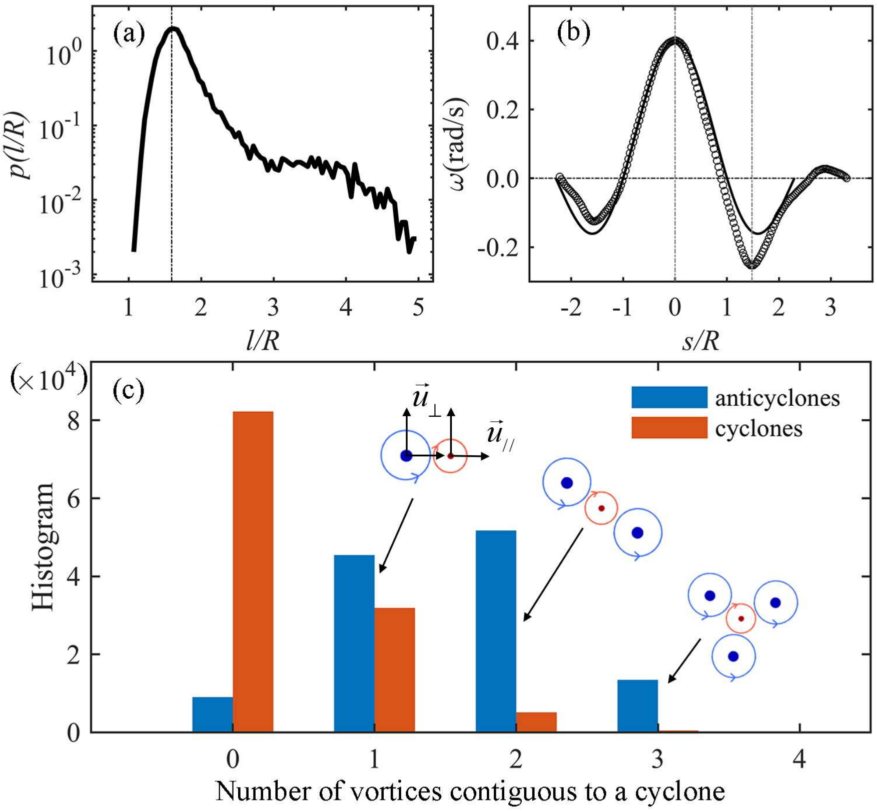

Supplementary Figure 8: Statistical data of vortex distributions. (a) Probability density function $p(l/R)$ of the distance l between an anticyclone and its neighboring cyclones within a cluster, normalized by the radius R of the anticyclone. The vertical dashed line shows a maximum of $p(l/R)$ at $l{=}l_{\mathrm{m}}{=}cR$. (b) Vorticity profile $\omega(s/R)$ of an opposite-sign vortex pair along the centerline s. The anticyclonic radius R is defined by the first zero of $\omega(s/R)$ from the vortex center. Open circles: experimental data. Solid line: the scaled zeroth-order Bessel functions: $J_0(ks/R)\omega(0)/J_0(0)$, where k=2.405 is the first zero of J_0. The two vertical dashed lines indicate the centers of the anticyclone and the cyclone at s_1=0 and $s_2{=}cR$. (c) Histogram of the numbers of anticyclones (blue) and cyclones (red) that are contiguous to one cyclone. Insets: schematic of three clustering modes. All data are for Ra=3×10^7, Fr=0.27, Ra/Ra$_{\mathrm{c}}$=1.97 and Γ=3.8.

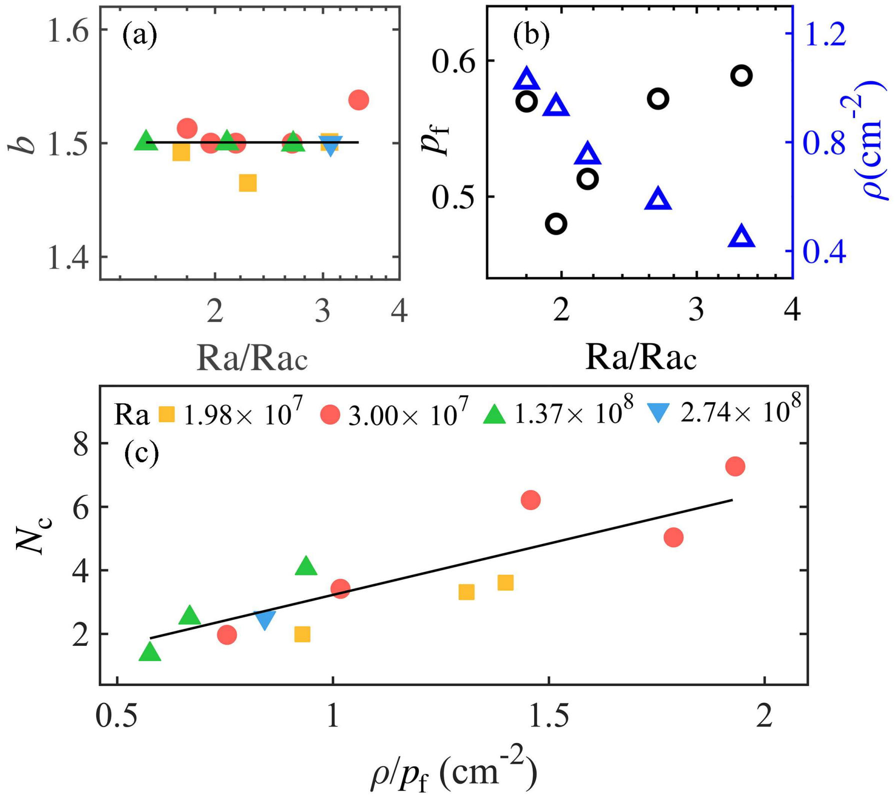

Supplementary Figure 9: Statistical properties of vortex clusters. (a) The power exponent b as a function of $\mathrm{Ra}/\mathrm{Ra}_c$ for various Ra and Γ. The black line presents the mean $\langle b \rangle$=1.50 for all data. Symbols are defined in (c). (b) The separation rate p_f (black circle) and the vortex population density ρ (blue triangle) as functions of $\mathrm{Ra}/\mathrm{Ra}_c$. p_f is determined by the fraction of vortices separated from a cluster after a time interval of 4 seconds. Results for Ra=3.0×10^7 and Γ=3.8. (c) The cutoff size N_c of clusters plotted against the ratio of ρ over p_f for various Ra and Γ. The black line presents a fitting: N_c=$3.23\rho/p_f$ [cm^{-2}]. Triangles: data for Γ=2; squares and circles: data for Γ=3.8.

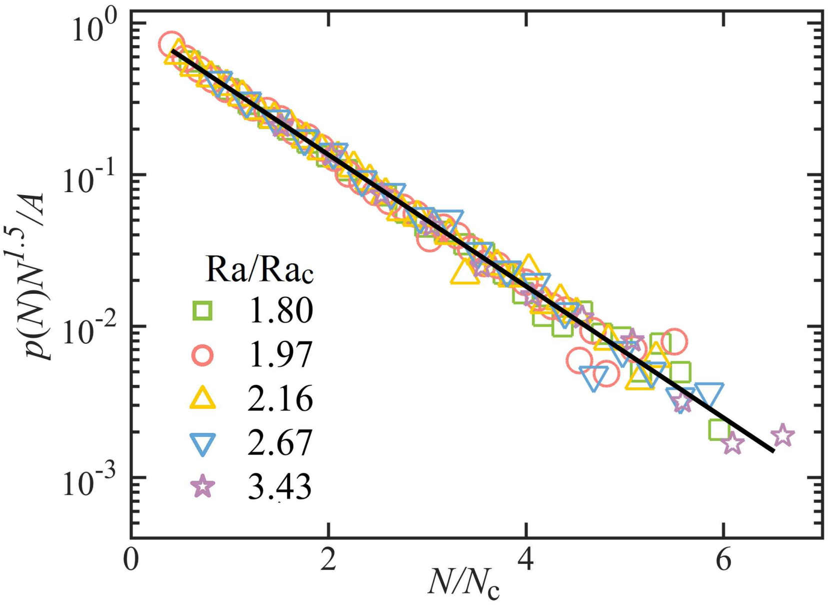

Supplementary Figure 10: Compensated plot of size distribution of vortex clusters. The rescaled data $p(N)N^{1.5}/A$ is shown in a semi-log frame as a function of N/N_c. The solid line represents an exponential function. Data for Ra=3.0×10^7.

Supplementary Table 1: Parameters for all experimental runs at fixed Pr=4.38. Results for Γ=3.8, 2.0 are from convection cells with H=6.3, 12.0cm, respectively.

10^{-7}Ra	10^5Ek	Ra/Ra$_c$	Fr	Γ	10^{-7}Ra	10^5Ek	Ra/Ra$_c$	Fr	Γ
2.0	1.79	1.30	0.272	3.8	6.0	1.79	3.94	0.272	3.8
2.0	1.92	1.43	0.237	3.8	6.0	2.24	5.34	0.174	3.8
2.0	2.24	1.76	0.174	3.8	6.0	2.68	6.86	0.121	3.8
2.0	2.68	2.26	0.121	3.8	6.0	3.36	9.32	0.077	3.8
2.0	3.36	3.07	0.077	3.8	6.0	4.47	13.9	0.044	3.8
2.0	4.47	4.57	0.044	3.8	6.0	5.37	17.8	0.030	3.8
2.0	5.37	5.87	0.030	3.8	6.0	8.95	36.1	0.011	3.8
2.0	13.4	20.9	0.005	3.8	6.0	13.4	63.3	0.005	3.8
2.0	26.8	54.8	0.001	3.8	14	0.493	1.54	0.272	2.0
3.0	1.68	1.80	0.309	3.8	14	0.617	2.09	0.174	2.0
3.0	1.79	1.97	0.272	3.8	14	0.740	2.68	0.121	2.0
3.0	1.92	2.16	0.237	3.8	14	0.925	3.64	0.077	2.0
3.0	2.24	2.67	0.174	3.8	14	1.23	5.39	0.044	2.0
3.0	2.68	3.43	0.121	3.8	14	1.48	6.91	0.030	2.0
3.0	3.36	4.66	0.077	3.8	27	0.493	3.09	0.272	2.0
3.0	4.47	6.93	0.044	3.8	27	0.617	4.18	0.174	2.0
3.0	5.37	8.90	0.030	3.8	27	0.740	5.36	0.121	2.0
3.0	8.95	18.0	0.011	3.8	27	0.925	7.27	0.077	2.0
3.0	13.4	31.6	0.005	3.8	27	1.23	10.8	0.044	2.0
3.0	26.8	83.1	0.001	3.8					

Supplementary Table 2: Parameters for all DNS cases at fixed Pr=4.38 and Ra=2.0×10^7.

10^{-7}Ra	10^5Ek	Ra/Ra$_c$	Fr	Γ	10^{-7}Ra	10^5Ek	Ra/Ra$_c$	Fr	Γ
2.0	1.78	1.30	0.270	4.0	2.0	1.78	1.30	0	4.0
2.0	1.90	1.42	0.240	4.0	2.0	1.90	1.42	0	4.0
2.0	2.22	1.76	0.174	4.0	2.0	2.22	1.76	0	4.0
2.0	2.66	2.26	0.120	4.0	2.0	2.66	2.26	0	4.0
2.0	3.33	3.07	0.077	4.0	2.0	3.33	3.07	0	4.0
2.0	3.67	3.51	0.064	4.0	2.0	3.67	3.51	0	4.0
2.0	4.44	4.57	0.040	4.0	2.0	4.44	4.57	0	4.0
2.0	5.41	5.99	0.029	4.0	2.0	5.41	5.99	0	4.0
2.0	7.00	8.55	0.017	4.0	2.0	9.18	12.4	0	4.0
2.0	9.18	12.4	0.010	4.0					
2.0	12.1	18.3	0.006	4.0					
2.0	18.2	32.2	0.003	4.0					
2.0	26.6	54.7	0.001	4.0					

Supplementary Note 1 — Phase diagram of the present study

We show in the Supplementary Fig. 1 the phase diagram of rotating convection in parameters of Ek and Ra/Ra_c. The parameter range of the present experimental and numerical studies are presented. Overall our data cover the range ($4.9\times10^{-6}\leq Ek\leq 2.7\times10^{-4}$, $1.3\leq Ra/Ra_c\leq 166$), and span the flow regimes of Buoyancy-dominated convection, Plumes/Geostrophic Turbulence(GT), Columns and Cells (Not turbulent) [1, 2, 3].

Supplementary Note 2 — Method of vortex identification and tracking

We identify the vortices through a two-dimensional Q-criterion, considering the quantity [5]

$$Q = (Tr\mathbf{A})^2 - 4det\mathbf{A}, \tag{1}$$

with the velocity gradient tensor $\mathbf{A}=[\partial(v_x, v_y)/\partial(x, y)]$. A connected region satisfying $Q<-Q_{\mathrm{std}}$ is defined as a vortex with Q_{std} being the standard deviation of Q over the cross-section of the sample. The vortex center is defined as the minimum of Q within each vortex region. We observed that the long-lived columnar vortices sustained by the gravitational buoyancy have on average a lifetime much larger than the timescale of vorticity diffusion $\tau_v=r_v^2/\nu\approx 10s$ (r_v is the mean radius of the vortices). For this reason, vortex trajectories with sufficient length can be traced as indicated in Fig. 1 of the main paper. In practice, the following approach is adopted in tracking vortices in our analysis. In a previous frame, for example, vortex A is identified and we determine its center position and the distance to its nearest neighboring vortex $d_{\min}$ (Supplementary Fig. 2a). In the next frame (Supplementary Fig. 2b), vortex B found at a nearest position to that of vortex A is considered as the same vortex if the following requirement is met: the distance between the center of vortex A and B satisfies $d_{\mathrm{AB}}<\beta d_{\min}$. The parameter β=0.3 is chosen based on the statistical data of the mean vortex speed to ensure that the displacements of most vortices between two successive frames are within $\beta d_{\min}$. The trajectory of a vortex is thus obtained when positions of the same vortex are linked in a time sequence. Vortices that are not identified in a prior frame are considered as newly-born vortices.

Supplementary Note 3 — Theoretical modeling of the centrifugal vortex motion

Under strong rotations the centrifugal force drives cold (warm) fluid radially away from (towards) the rotation axis. We consider a set of Langevin-type equations that describe the radial motion of the vortices under the centrifugation effect:

$$m\ddot{r} + \eta\dot{r} \pm m\alpha\delta T\Omega^2 r = \xi(t). \tag{2}$$

Here the vortices are modeled as inertial coherent structures [6] that undergo viscous damping, centrifugal buoyancy and the random forcing from the background flows. Since the Coriolis force acts on moving fluids both inside and outside the vortex structures, we assume that its overall effect on the radial vortex motion is secondary compared to that from the centrifugal buoyancy. In Supplementary Eq. 2 m is the inertial mass of the vortex. η is the friction coefficient. The centrifugal force is expressed in terms of $\pm m\alpha\delta T\Omega^2 r$ for the relatively warm cyclones (plus sign) and the cold anticyclones (minus sign), with δT being the temperature difference between the vortex and the background fluid. Thus $m\alpha\delta T$ presents the mass difference between the vortex and the background fluid of the same volume. $\Omega^2 r$ is the centrifugal acceleration. We define the relaxation timescale of damping $\tau=m/\eta$ and the centrifugal coefficient $\zeta=\alpha\delta T\Omega^2$ to obtain

$$\ddot{r} + \dot{r}/\tau \pm \zeta r = \xi^*(t) \tag{3}$$

with $\xi^*(t)=\xi(t)/m$. The background turbulent fluctuations are modeled by a δ-correlated Gaussian noise

term:

$$\langle \xi^*(t) \rangle = 0, \tag{4a}$$

$$\langle \xi^*(t)\xi^*(t+\Delta t) \rangle = \delta(\Delta t) D/\tau^2, \tag{4b}$$

where D is the diffusivity that measures the strength of the background fluctuations. In Supplementary Eqs. 2-4 the radial motion of the cyclones (anticyclones) are modeled as randomly forced normal (inverted) harmonic oscillators, with the parabolic potentials created by the centrifugal force. In the slow rotating limit, the centrifugal coefficient ζ approaches zero and the model reduces to that for the classical Brownian-motion.

In the presence of the centrifugal force, the horizontal translational invariance of the vortex motion is lost and the motion of each vortex is dependent on its radial position as well as its previous dynamical status (such as previous velocity and acceleration), leading to a non-analytical solution of the mean-square-displacement. Here we present a theoretical approach to derive the general solutions of the first and second moments of the vortex displacement from Supplementary Eqs. 3 and 4. Defining a variable $\dot{p}$=$\ddot{r}$+$(1/\tau - \lambda)\dot{r}$, and consider the equation of motion for anticyclones, we may rewrite Supplementary Eq. 3 as a combination of two equations for (r, p):

$$\dot{p} + \lambda p = \xi^*(t), \tag{5a}$$

$$\dot{r} - \zeta r/\lambda = p. \tag{5b}$$

The coefficient λ satisfies the equation $\lambda^2 - \lambda/\tau - \zeta = 0$ that has two roots, $\lambda_{1,2} = \pm\sqrt{\zeta + 1/(4\tau^2)} + 1/2\tau$. They are used as control variables of the dynamical system (Supplementary Eq. 5) that replace ζ and τ. In our experimental system $|\zeta| \ll 1/(4\tau^2)$, yielding real values of $\lambda_{1,2}$, thus the vortices exhibit overdamping motions. Integration of Supplementary Eq. 5 yields the solutions:

$$p(t) = C_1 e^{-\lambda t} + \int_0^t e^{\lambda(s-t)} \xi^*(s) ds, \tag{6a}$$

$$r(t) = C_2 e^{\zeta/\lambda t} + \int_0^t e^{\zeta(t-s)/\lambda} p(s) ds. \tag{6b}$$

The coefficients C_1 and C_2 are dependent on initial conditions, i.e., $C_1 = -\lambda_2 r_0$ and $C_2 = r_0$. Without losing generality, we let $\lambda = \lambda_1$ and thus $\zeta/\lambda = -\lambda_2$. Substituting Supplementary Eq. 6a into 6b, and using the initial conditions $(r(t{=}0) = r_0, v(t{=}0) = 0)$, we obtain the general solution of $r(t)$:

$$r(t) = r_0 e^{-\lambda_2 t} + \frac{r_0 \lambda_2}{\lambda_1 - \lambda_2}(e^{-\lambda_2 t} - e^{-\lambda_1 t}) + \frac{1}{\lambda_2 - \lambda_1}\int_0^t (e^{\lambda_1(s-t)} - e^{\lambda_2(s-t)}) \xi^*(s) ds. \tag{7}$$

Since the noise term $\xi^*(t)$ has a zero mean (Supplementary Eq. 4a), we take the average of r/r_0 over the trajectory ensemble to derive the expressions of first moment of the radial displacement:

$$\langle r/r_0 \rangle_\xi = \frac{\lambda_1}{\lambda_1 - \lambda_2} e^{-\lambda_2 t} - \frac{\lambda_2}{\lambda_1 - \lambda_2} e^{-\lambda_1 t}. \tag{8}$$

Here $\langle ... \rangle_\xi$ presents the average with respect to the distribution of the stochastic variable $\xi^*(t)$, or the trajectory-assemble average. Following the solution of $r(t)$ (Supplementary Eq. 7) and using the fact that the noise term $\xi^*(t)$ is δ-correlated (Supplementary Eq. 4b), we obtain the second order moment of the

radial displacement:

$$\langle[r(t)-\langle r(t)\rangle_\xi]^2\rangle_\xi = \frac{1}{(\lambda_2-\lambda_1)^2}\int_0^t\int_0^t (e^{\lambda_1(s_1-t)}-e^{\lambda_2(s_1-t)})(e^{\lambda_1(s_2-t)}-e^{\lambda_2(s_2-t)})\langle\xi^*(s_1)\xi^*(s_2)\rangle ds_1 ds_2$$
$$= \frac{D}{\tau^2(\lambda_2-\lambda_1)^2}[\frac{1-e^{-2\lambda_1 t}}{2\lambda_1}+\frac{1-e^{-2\lambda_2 t}}{2\lambda_2}-\frac{2-2e^{-(\lambda_1+\lambda_2)t}}{\lambda_1+\lambda_2}]. \tag{9}$$

The dynamical system that governs the cyclonic motion reads

$$\dot{p}+\lambda p=\xi^*(t), \tag{10a}$$

$$\dot{r}+\zeta r/\lambda = p, \tag{10b}$$

with the λ-equation given by: $\lambda^2-\lambda/\tau+\zeta=0$, and the two roots being $\lambda_{1,2}=\pm\sqrt{-\zeta+1/(4\tau^2)}+1/(2\tau)$. Following the same approach one obtains solutions for the first and second moment of displacements of cyclones, which are exactly in the same expressions as Supplementary Eq. 8 and Eq. 9 respectively. Supplementary Equations 8 and 9 show that the first and second moments of the vortex displacements are both sums of multiple exponential functions of time. In the large-time limit ($t\gg\tau$) both statistical moments asymptote to a single exponential function:

$$\langle r/r_0\rangle_\xi \approx \frac{\lambda_1}{\lambda_1-\lambda_2}e^{\pm\lambda^* t}, \tag{11}$$

and

$$\langle[r(t)-\langle r(t)\rangle_\xi]^2\rangle_\xi \approx \frac{D}{\tau^2(\lambda_2-\lambda_1)^2}[\frac{(\lambda_1-\lambda_2)^2}{2\lambda_1\lambda_2(\lambda_1+\lambda_2)}-\frac{e^{\pm 2\lambda^* t}}{2\lambda_2}]. \tag{12}$$

Here $\lambda^*=|1/(2\tau)-\sqrt{1/(4\tau^2)\pm\zeta}|$ is the fastest growth (or slowest decay) rate for anticyclones (cyclones), which represents the mobility of vortices in the context of centrifugal acceleration.
We show in Figs. 2a and 2b of the main paper our theoretical predictions of the first and second moments of the vortex displacements (Supplementary Eqs. 8 and 9) that are compared to the experimental data. The first moment of $\langle r/r_0\rangle_\xi$ represents the mean radial displacement of the vortices. The second moment, $\langle(r-\langle r\rangle_\xi)^2\rangle_\xi$, reveals the mean deviations of the vortex trajectories from their mean path. Notably the theoretical curves of $\langle(r-\langle r\rangle_\xi)^2\rangle_\xi$ for anticyclones take on the feature of superdiffusion at large times, as they increase steeply after an inflection point in a log-log plot, whereas this feature is absent for cyclones as the cyclonic data curves appear to level off. These predicted trends are in close agreement with the experimental data for all anticyclones, and for the cyclones outside the anomalous, inverse-centrifugation regime. The model is, however, incapable of explaining the inverse centrifugal motion of the cyclones in the anomalous regime. We provided their interpretations from the point view of collective vortex motion in the main paper. Supplementary Figure 3 shows results of the parameters of the model including the momentum relaxation time τ, the diffusivity D and the fastest growth (slowest decay) rate λ^* for anticyclones (cyclones). These parameters are determined through least-square fittings of the experimental data through Supplementary Eqs. 8 and 9, minimizing the summation of the deviations from the theoretical predictions. Note that no fitting is made for the cyclonic motion in the anomalous regime.
From Supplementary Eq. 11 one obtains the assemble-average of the radial velocity u_r, normalized by the initial vortex radial position r_0:

$$\langle u_r/r_0\rangle_\xi \approx \frac{\lambda_1\lambda^*}{\lambda_1-\lambda_2}e^{\pm\lambda^* t} \approx \lambda^*\langle r/r_0\rangle_\xi. \tag{13}$$

which implies that for a given r_0, $\langle u_r\rangle_\xi$ is proportional to $\langle r\rangle_\xi$ in large time scales ($t\gg\tau$). However, there exists no analytic solution of $\langle u_r\rangle_\xi(r)$ in general for arbitrary initial vortex positions r_0.

In the slow rotating limit ($\zeta \approx 0$), Supplementary Equations 3 and 4 are reduced to the classical Langevin equations for Brownian motion and the MSD of the vortices are given by

$$\langle (r(t+dt) - r(t))^2 \rangle = 2D[dt - \tau(1 - e^{-dt/\tau})]. \tag{14}$$

For experimental data in the randomly-diffusive regime we apply Supplementary Eq. 14 to determine τ and D. These results are shown together in Supplementary Fig. 3.

Supplementary Note 4 — Statistics of vortex motions in both the upper- and lower-half of the fluid layer

Figure 2 in the main paper presents experimental results of the first and second moments of the vortex displacements measured in the lower half of the fluid layer. Here we provide these statistical results obtained through DNS in both the upper and lower fluid heights.

(i) Vortex motion in the strongly rotating, In the strongly rotating, inverse-centrifugal regime.
In this flow regime the vortices appear as columnar vortices (e.g. left inset in Fig. 3 of the main paper), with their horizontal motions being similar at all fluid heights. We show in Supplementary Fig. 4 the first and the second moments of the vortex radial displacements in two vertical positions z/H=0.2 and z/H=0.8. One sees that for the two vertical positions the statistical results of both the first and the second moments are nearly identical. They reveal the outward motion of the cold vortices and the abnormal outward motion of the warm vortices. (Note that the statistical time for cold vortices are longer than the warm ones because they are relatively stronger and possess on average a longer lifetime.) The cold vortex motion observed in both vertical positions are found to be well described by our theoretical model (see Supplementary Note 3). Panels (c) and (f) of Supplementary Fig. 4 present respectively trajectories of a cold and a warm vortex, and show that the vortex trajectories measured at the two fluid heights for the same vortex indeed match approximately with each other.

(ii) Vortex motion in the centrifugation-influenced regime with relatively slow rotations.
In this flow regime the vortex structures are strongly dependent on the fluid height, the cold (warm) vortices forming at the top (bottom) boundary layer do not reach the opposite side of the fluid layer (see the right inset in Fig. 3 of the main paper). For these reasons we observe less cold (warm) vortices in the lower (upper) fluid level and vise verse in this regime. Supplementary Fig. 5 shows results of the first and second moments of the vortex radial displacements at two fluid depths. As expected the statistical data obtained for warm vortices at the upper fluid level (z/H=0.8) are less than that at the lower level (z/H=0.2), and vise verse for the cold vortices. However, the vortices still exhibit clearly motions in the same direction in both the upper and the lower fluid layers. In the short-time domain where data of both types of vortices are available, their statistical moments overlap with each other, indicating the same physical mechanism that governs their horizontal motion.

Supplementary Note 5 — Comparisons between the radial velocity of the vortices and the meridional circulation

Previous studies in rapidly rotating RBC revealed a meridional circulation driven by the centrifugal buoyancy in which cool denser fluid is moving radially outward near the top boundary and warm lighter fluid moving inward near the bottom boundary (e.g., [7, 8, 9] Our numerical data show clearly the existence of this axisymmetric meridional circulation in flow regimes with $\mathrm{Ra}/\mathrm{Ra_c} \leq 10$. The radial component of this

circulation is found to be dependent on the radial position r and is in agreement with previous studies. In Supplementary Fig. 6 we show the azimuthal- and time-averaged radial fluid velocity $\langle v_r \rangle$ as functions of $\mathrm{Ra}/\mathrm{Ra_c}$. Here $\langle v_r \rangle$ is measured at $r=d/4$, at distances $z=\mathrm{Ek}^{1/2}H$ from the top and bottom plates, where the magnitude of $\langle v_r \rangle$ is maximum. Results of $\langle v_r \rangle$ are compared with the mean radial velocity $\langle u_r \rangle$ of both the warm (upwelling) and cold (downwelling) vortices. We see that overall $\langle v_r \rangle$ and $\langle u_r \rangle$ show very different dependences of $\mathrm{Ra}/\mathrm{Ra_c}$. The mean radial velocity of cold vortices $\langle u_r^c \rangle$ is larger than the meridional circulation velocity $\langle v_r^t \rangle$ near the top boundary. When the centrifugal effect is dominant ($\mathrm{Ra}/\mathrm{Ra_c}\approx 2.0$), $\langle u_r^c \rangle$ becomes 4-5 times in magnitude greater than $\langle v_r^t \rangle$. For the radial velocity of warm vortices, we see that in the inverse-centrifugal regime (Regime II, $1.6\leq \mathrm{Ra}/\mathrm{Ra_c}\leq 4.0$) $\langle u_r^w \rangle$ exhibits outward (inverse-centrifugal) motion as explained in the main paper, which is opposite to the circulation direction $\langle v_r^b \rangle$ near the bottom boundary. Our data here thus provide direct evidence that the centrifugal circulation is not the origin for the observed horizontal motion of the vortices.

Meanwhile, since the centrifugal circulation drives radial fluid motion inside the viscous boundary layers (BLs), we believe that this secondary flow may alter the viscous drag acting on the vortices during their horizontal motion. For instance, the outward circulation near the top boundary reduces (enhances) the viscous damping for the outward cold vortex (inward warm vortex) motion, and viceversa near the bottom boundary. In our theoretical model, the effect of viscous damping (mainly from the viscous BLs) for vortex motion, is parametrized by a relaxation timescale τ accounting for the damping effect, which is allowed to vary as functions of $\mathrm{Ra}/\mathrm{Ra_c}$ independently for cyclones and anticyclones (see Supplementary Fig. 3).

Supplementary Note 6 — Flow field symmetry when the centrifugal buoyancy is absent

We present in Supplementary Fig. 7 numerical results of the temperature and vorticity fields when the centrifugal buoyancy is absent. These data are obtained at a fluid height $z=0.2H$ of a cylindrical cell for $\mathrm{Ra}=2.0\times 10^7$ and $\mathrm{Ra}/\mathrm{Ra_c}=2.26$, i. e., with the same numerical settings as those shown in Fig. 3b and 3c in the main text except that the centrifugal buoyancy is switched off. Supplementary Fig. 7a shows that the background fluid temperature T_c is close to T_m, the mean of the top and bottom boundary temperature (T_c is slightly larger than T_m because the data are taken at the lower half of the fluid layer, $z=0.2H$). Moreover, in the bulk region ($r\leq 0.4d$) the mean temperatures of cold and warm vortices locate nearly symmetrically on the lower and upper sides of T_c, which contrasts strongly with the case when centrifugal buoyancy is present. Supplementary Fig. 7b shows that the ratios of vortex temperature γ_ω and vorticity γ_T are identical and close to unity in the bulk, again, in strong contrast to the case of non-zero centrifugal buoyancy (see Fig. 3b and 3c in the main text). Distribution of the temperature anomaly $\delta T/\Delta T$ are shown in Supplementary Fig. 7c. In the near-sidewall region ($0.4d\leq r\leq 0.5d$) large fluctuations in the temperature field appear owing to the perturbations of the boundary flows, which gives rise to the fluctuations of the means of the temperature and vorticity shown in Supplementary Figs. 7a and 7b.

Supplementary Note 7 — Hydrodynamic interaction of an opposite-signed vortex pair

As illustrated in Fig. 4 of the main text, adjacent vortices within a cluster tend to move in the same direction. This phenomenon of highly correlated vortex motion is observed in rotation-dominated convection where the flow fields are largely constrained by the strong rotations. Asymptotic theory of rotating RBC requires the vortex structure to be columnar and axis-symmetric [10]. The theory predicts that for a single vortex the radial profiles of the vertical vorticity can be expressed by the zeroth-order Bessel function J_0, with a shielded structure of opposite vorticity forming near the edge of the vortex core. These properties of vortex structure are captured in high-resolution flow field measurements [11].

Figure 4 in the main text suggests that in this flow regime the clustered vortices are densely populated, with each vortex surrounded more likely by counter-rotating vortices. In Supplementary Fig. 8a we show, inside a cluster, the probability density function $p(l/R)$ of the distance l between an anticyclone and its neighboring cyclones, normalized by the anticyclonic radius R. One sees that $p(l/R)$ has an apparent maximum at l_m/R=1.593. Note that the ratio is less than 2 because the size of cyclones is on average less than that of anticyclones. We find that l_m/R equals to the ratio of the first minimum and the first zero of the zeroth-order Bessel function J_0 (see interpretations below). Since $p(l/R)$ represents the probability of finding cyclones at a distance l from an anticyclone, it reflects the interactions of adjacent counter-rotating vortices. To obtain some insight into the prominent maximum of $p(l/R)$, we show in Supplementary Fig. 8b an example of the vorticity profile $\omega(s/R)$ along the connecting line (the centerline) s of two opposite-signed, neighboring vortices. We see that within the core region ($s \leq R$) the vorticity profile $\omega(s/R)$ is broadly in agreement with the zeroth-order Bessel functions J_0. Moreover, the cyclone is located approximately at a distance $s = l_\mathrm{m}$ from the adjacent anticyclonic center, where J_0 reaches a first minimum. Such a pair-wise vorticity profile is commonly observed within vortex clusters. We provide the following two interpretations for the most probable vortex separation l_m. First of all, one sees in Supplementary Fig. 8b that the most probable vortex separation l_m is such that it enables the vorticity field within the core region for both vortices to attain the highest level of axis-symmetry, so that it is compatible with the zeroth-order Bessel function J_0. This property of flow symmetry is required in the theories of rapidly rotating RBC [10, 12]. Secondary, the radial fluid velocity $v_r(s/R)$ of the anticyclone, given by the first-order Bessel function J_1 [10, 12], crosses zero with a negative gradient at $s = l_\mathrm{m}$ (thereby $v_r > 0$ for $s < l_\mathrm{m}$ and $v_r < 0$ for $s > l_\mathrm{m}$). Thus l_m is a radial position of stable local equilibrium for nearby meandering cyclones. This explains the prominent peak of $p(l/R)$. The shielded structures for both vortices prevents them from further aggregation, leading to the fast decay of $p(l/R)$ for $l < l_\mathrm{m}$.

We next show that two adjacent counter-rotating vortices have similar velocities of horizontal motion. Since the two vortices maintain a constant separation l_m from each other, if one vortex moves along their centerline at velocity $\vec{u}_\parallel$, either due to inertial motion or disturbance of ambient flows, its counterpart vortex moves with the same velocity (see inset of Supplementary Fig. 8c). Their velocity components $\vec{u}_\perp$ perpendicular to the centerline, determined by the azimuthal fluid velocity v_θ of the counterpart vortex, are similar in direction [13]. For a more comprehensive understanding of the collective motion of the interacting vortices, we examine further the various modes of vortex clustering. Supplementary Fig. 8c shows a histogram for the number of vortices that are adjacent to one cyclone, i.e., the vortices involved in direct interactions with the cyclone. We find that a cyclone can be contiguous to different number of anticyclones or cyclones, and most probably it is in direct contact with two neighboring anticyclones. In this most probable mode, the overall fluid velocity at the cyclonic center, induced by the two neighboring anticyclones, is close to zero, assuming that the two anticyclones are circular and equal in strength. Since the net centrifugal force acting on the three vortices is mainly due to the two strong anticyclones, they move outwardly with approximately the same speed, keeping the distance between the cyclone and each anticyclone nearly constant (i.e., $l \approx l_\mathrm{m}$). Similar collective behavior of the vortices is observed in other clustering modes, which can be interpreted along the line of the aforementioned vortex interaction.

Supplementary Note 8 — Physical mechanism of vortex aggregation and separation

As discussed in the main text, we observed stationary cluster-size distribution that can be expressed as $p(N) = AN^{-b}e^{-N/N_\mathrm{c}}$ for various rotation rates with Ra=3.0×10^7 and Γ=3.8. Such an exponentially truncated power function is found to describe well results of $p(N)$ for different Ra and Γ. Moreover, we find that the power-law exponent b=1.50$\pm$0.04 remains a constant in all vortex-cluster measurements, irrespective of Ek, Ra and Γ (see Supplementary Fig. 9a). Such a robust three-half power scaling of $p(N)$ for small N, predicted in previous theoretical models of grouping dynamics [14, 15, 16] suggests that

self-aggregation of adjacent vortices is the underlying physics responsible for the formation of the vortex clusters. In Supplementary Movie 3, one sees that in the centrifugation-dominated flow regime, both the clustered strong anticyclones and weak cyclones move outward collectively. Along the path of their outward motion they encounter frequently other isolated vortices. If the distance from the isolated vortex to the nearest opposite-signed vortex in the cluster becomes close to the critical separation l_m (defined in Supplementary Note 7), the initially isolated vortex often self-aggregates into the cluster and joins in the collective motion. Our interpretation of this aggregation phenomenon is as follows: the hydrodynamic vortex-pair interaction (see discussions of Supplementary Fig. 8b) effectively results in an attractive potential, reflected by $p(l/R)$ in Supplementary Fig. 8a, for the motion of isolated vortices surrounding the clustered vortices. As discussed in Supplementary Note 7, during their stochastic motion the isolated vortices have a large probability of converging to the stable position with a radial distance l=l_m from the nearest clustered vortices. It is this individual behavior of vortex aggregation that leads to the power-law cluster-size distribution $p(N)$ for small N.

In contrast to the vortex aggregation process, vortices may often separate from clusters, owing to the instability of the cluster structures when disturbed by the turbulent ambient flows. The statistical properties are therefore a result of the continuous process of both aggregation and separating of vortices that maintains a stationary cluster-size distribution $p(N)$. It has been reported in previous studies [16, 17] that a sufficient large separation rate of the entities from the clusters may result in an exponential decay of the $p(N)$ for large N. Supplementary Figure 9b shows the separation rate p_f of vortices from a cluster as a function of Ra/Ra$_\mathrm{c}$. We see that p_f has an apparent minimum at Ra/Ra$_\mathrm{c}$=1.97. It corresponds to the situation in which the centrifugal effect is dominant and the vorticity ratio γ_ω is maximum (see Fig. 3 in the main text). This result implies that when the vorticity field reaches the highest level of asymmetry and the anticyclonic flows is the dominant factor in vortex interactions, the cluster structure is most stable against fragmentation. Analogous to other biological systems [16], we find that the characteristic size N_c of the clusters is proportional to the ratio of the vortex population density ρ over the separation rate p_f (Supplementary Fig. 9c). Although the vortex population density ρ decreases with increasing Ra/Ra$_\mathrm{c}$ (shown in Supplementary Fig. 9b), N_c has a maximum at Ra/Ra$_\mathrm{c}$=1.97 (reported in Fig. 5a of the main text), because of the prominent minimum of p_f at this parameter condition.

In Supplementary Fig. 10 we show the size distribution of vortex clusters $p(N)$, compensated by a power function $N^{1.5}$. One sees that the rescaled data $p(N)N^{1.5}/A$ for various Ra/Ra$_\mathrm{c}$ collapse onto a straight line in a semi-log plot, indicating that $p(N)$ is well described by the product of a three-half power function and an exponential function.

Supplementary Note 9 — Correlation function of vortex velocity fluctuations

The velocity fluctuation correlation function of vortices within one cluster is determined by the velocity fluctuation $\vec{u'}_i$ of each vortex with respect to the mean velocity of all vortices in the cluster

$$C(l) = \frac{\sum_{ij}(\vec{u'}_i(\vec{r}_i + \vec{l})\cdot\vec{u'}_j(\vec{r}_j)\delta(l - l_{ij}))}{C_0\cdot\sum_{ij}\delta(l - l_{ij})}. \tag{15}$$

Here $\delta(l - l_{ij})$ is a Dirac function selecting pairs of vortices separated by distance l, $\sum_{ij}$ represents the summation over all possible vortex pairs within a cluster and C_0 is a normalization factor setting $C(l{=}0)$=1. To ensure sufficient statistics, we take average of the vortex velocity data over all clusters of size N when calculating $C(l)$.

Results of $C(l)$ for clusters with various size N are shown in Figs. 5c and 5d in the main paper. We find

that the obtained correlation functions can be fitted by a stretched exponential function [18]

$$C(l) = (1+a)e^{(-c_1 l)^{c_2}} - a, \tag{16}$$

with three fitting parameters (a, c_1, c_2). Figure 5c in the main paper presents the following fitting functions of $C(l/H)$ for N=6, 7, 12, respectively:

$$C(l/H) = 1.45e^{-(l/H/0.077)^{0.85}} - 0.45, \tag{17}$$

$$C(l/H) = 1.45e^{-(l/H/0.115)^{0.85}} - 0.45, \tag{18}$$

$$C(l/H) = 1.45e^{-(l/H/0.146)^{0.85}} - 0.45. \tag{19}$$

Figure 5d in the main paper shows that, when the separation distance l of each vortex pair is normalized by the cluster length scale L, all the obtained $C(l/L)$ collapse onto a single curve that is independent of the cluster size N:

$$C(l/L) = 1.45e^{-(l/L/0.245)^{0.85}} - 0.45. \tag{20}$$

Supplementary Refereces

[1] Julien, K. & Rubio, A. M. & Grooms, I. & Knobloch, E. Statistical and physical balances in low Rossby number Rayleigh-Bénard convection. *Geophys. Astrophys. Fluid. Dyn.* **106**, 392–428 (2012).

[2] Aurnou, J. M. *et al.* Rotating convective turbulence in Earth and planetary cores. *Phys. Earth Planet. Inter.* **246**, 52–71 (2015).

[3] Kunnen, R. P. J. The geostrophic regime of rapidly rotating turbulent convection. *J. Turbul.* (2021). DOI: 10.1080/14685248.2021.1876877.

[4] Lu, H.-Y. & Ding, G.-Y. & Shi, J.-Q. & Xia, K.-Q. & Zhong, J.-Q. Heat transport scaling and transition in geostrophic rotating convection with varying aspect ratio. *Phys. Rev. Fluids* **6**, L071501 (2021).

[5] Vorobieff, P. & Ecke, R. E. Turbulent rotating convection: an experimental study. *J. Fluid Mech.* **458**, 191–218 (2002).

[6] Chong, K. L. *et al.* Vortices as Brownian particles in turbulent flows. *Sci. Adv.* **6**, eaaz1110 (2020).

[7] Homsy, G. M. & Hudson, J. L. Centrifugally driven thermal convection in a rotating cylinder. *J. Fluid Mech.* **35**, 33–52 (1969).

[8] Hart, J. E. & Ohlsen, D. R. On the thermal offset in turbulent rotating convection. *Phys. Fluids* **11**, 2101–2107 (1999).

[9] Homsy, G. M. & Hudson, J. L. Rotating convection with centrifugal buoyancy: Numerical predictions for laboratory experiments. *Phys. Rev. Fluids* **4**, 073501 (2019).

[10] Grooms, I., Julien, K., Weiss, J. B. & Knobloch, E. Model of convective Taylor columns in rotating Rayleigh-Bénard convection. *Phys. Rev. Lett.* **104**, 224501 (2010).

[11] Shi, J.-Q., Lu, H.-Y., Ding, S.-S. & Zhong, J.-Q. Fine vortex structure and flow transition to the geostrophic regime in rotating Rayleigh-Bénard convection. *Phys. Rev. Fluids* **5**, 011501 (2020).

[12] Portegies, J. W., Kunnen, R. P. J., van Heijst, G. J. F. & Molenaar, J. A model for vortical plumes in rotating convection. *Phys. Fluids* **20**, 066602 (2008).

[13] Leweke, T., Le Dizés, S. & Williamson, C. H. K. Dynamics and instabilities of vortex pairs. *Annu. Rev. Fluid Mech.* **48**, 507–541 (2016).

[14] Takayasu, H., Nishikawa, I. & Tasaki, H. Power-law mass-distribution of aggregation systems with injection. *Phys. Rev. A* **37**, 3110-3117 (1988).

[15] Takayasu, H., Steady-state distribution of generalized aggregation system with injection. *Phys. Rev. Lett.* **63**, 2563-2565 (1989).

[16] Bonabeau, E., Dagorn, L. & Freon, P. Scaling in animal group-size distributions. *Proc. Natl. Acad. Sci. USA* **96**, 4472–4477 (1999).

[17] Niwa, H.-S. Power-law versus exponential distributions of animal group sizes. *J. Theor. Biol.* **224**, 451–457 (2003).

[18] Chen, X., Dong, X., Be'er, A., Swinney, H. L. & Zhang, H. P. Scale-invariant correlations in dynamic bacterial clusters. *Phys. Rev. Lett.* **108**, 148101 (2012).
