# Supplementary material for: Inverse centrifugal effect induced by collective motion of vortices in rotating thermal convection
Source: Nat Commun. 2021 Sep 22;12:5585. doi: 10.1038/s41467-021-25838-3 (PMC8458392; doi:10.1038/s41467-021-25838-3)
Supplement: Supplementary file 3 — Description of Additional Supplementary Files [file 41467_2021_25838_MOESM3_ESM.docx]

Description of Additional Supplementary Files

Title: Supplementary Movie 1

Description: Experimental movie corresponding to Fig. 1a in the main paper. The movie shows the time lapse of the vorticity Įeld over a horizontal cross-section of the cell at z=H/4. Results for Ra=3.0×107 , Fr=0.03 and Ra/Rac=8.90. The movie runs at 50 times real speed.

Title: Supplementary Movie 2

Description: Experimental movie corresponding to Fig. 1c in the main paper. Results over a horizontal cross-section of the cell at z=H/4 for Ra=3.0×107 , Fr=0.27 and Ra/Rac=1.97. The movie runs at 50 times real speed.

Title: Supplementary Movie 3

Description: Experimental movie corresponding to Fig. 4 in the main paper. The movie shows the time lapse of the Q-value Įeld over a horizontal cross-section of
